# Effect of Running Speed and Leg Prostheses on Mediolateral Foot Placement and Its Variability

**DOI:** 10.1371/journal.pone.0115637

**Published:** 2015-01-15

**Authors:** Christopher J. Arellano, William J. McDermott, Rodger Kram, Alena M. Grabowski

**Affiliations:** 1 Integrative Physiology Department, University of Colorado, Boulder, Colorado, United States of America; 2 Biomechanics Laboratory, The Orthopedic Specialty Hospital, Murray, Utah, United States of America; 3 Eastern Colorado Healthcare System, Denver, Colorado, United States of America; University of Manchester, UNITED KINGDOM

## Abstract

This study examined the effects of speed and leg prostheses on mediolateral (ML) foot placement and its variability in sprinters with and without transtibial amputations. We hypothesized that ML foot placement variability would: 1. increase with running speed up to maximum speed and 2. be symmetrical between the legs of non-amputee sprinters but asymmetrically greater for the affected leg of sprinters with a unilateral transtibial amputation. We measured the midline of the body (kinematic data) and center of pressure (kinetic data) in the ML direction while 12 non-amputee sprinters and 7 Paralympic sprinters with transtibial amputations (6 unilateral, 1 bilateral) ran across a range of speeds up to maximum speed on a high-speed force measuring treadmill. We quantified ML foot placement relative to the body’s midline and its variability. We interpret our results with respect to a hypothesized relation between ML foot placement variability and lateral balance. We infer that greater ML foot placement variability indicates greater challenges with maintaining lateral balance. In non-amputee sprinters, ML foot placement variability for each leg increased substantially and symmetrically across speed. In sprinters with a unilateral amputation, ML foot placement variability for the affected and unaffected leg also increased substantially, but was asymmetric across speeds. In general, ML foot placement variability for sprinters with a unilateral amputation was within the range observed in non-amputee sprinters. For the sprinter with bilateral amputations, both affected legs exhibited the greatest increase in ML foot placement variability with speed. Overall, we find that maintaining lateral balance becomes increasingly challenging at faster speeds up to maximum speed but was equally challenging for sprinters with and without a unilateral transtibial amputation. Finally, when compared to all other sprinters in our subject pool, maintaining lateral balance appears to be the most challenging for the Paralympic sprinter with bilateral transtibial amputations.

## Introduction

When running and sprinting, individuals with transtibial amputations face unique biomechanical constraints when using running-specific prostheses. When compared to the biological leg, the affected leg fitted with a running-specific prosthesis generates lower ground forces and exhibits less stiffness [1–3]. Additionally, using these passive-elastic leg prostheses likely challenges a person’s ability to maintain balance while running and sprinting because the design of running-specific prostheses primarily facilitates forward sagittal plane motion. Running-specific prostheses are passive devices made of carbon fiber that can only store and return elastic energy. In addition, running-specific prostheses have a fixed stiffness, whereas the stiffness of the human leg can be neurally modulated and thus non-amputees can adapt to changes in surface stiffness and speed [2, 4]. Further, unlike an intact human foot, running-specific prostheses cannot provide direct sensory information about ground contact and the distal end of the residual limb lacks the neural specialization that the plantar surface of the foot provides. As a final point, running-specific prostheses do not provide any proprioceptive feedback about “ankle” joint or foot position. In light of these observations, we were curious to explore how well sprinters can modulate foot placement from step-to-step when using running-specific prostheses.

Our recent studies of runners with two biological legs have demonstrated that adjusting step width from step-to-step is an effective strategy for maintaining lateral balance [5, 6]. We challenged a runners’ ability to maintain lateral balance by having them run with a range of unnaturally large step widths [5]. Running with non-preferred step widths increased step width variability and increased metabolic cost, thus increasing the effort to maintain lateral balance. In a follow up study [6], we reduced the muscular effort needed to maintain lateral balance during running by providing external lateral support with mechanical springs attached to a waist harness. Providing external lateral support decreased step width variability and decreased metabolic cost, thus reducing the effort to maintain lateral balance. Taken together, these studies suggest that mediolateral (ML) foot placement variability can be used as an indicator of lateral balance during running.

Although step width—the ML distance between the left and right foot during subsequent steps—provides a general measure of foot placement, it is insensitive to foot placement asymmetries that may exist between the legs of the same individual. Because runners with a unilateral transtibial amputation exhibit a variety of inter-limb biomechanical asymmetries [1, 7–11], we reasoned that measuring ML placement of each foot relative to the midline of the body, as opposed to step width, would be a more appropriate metric for elucidating any asymmetries that may exist between the individual legs. Thus, in the present study, we quantified ML foot placement relative to the body’s midline and its variability.

Our previous experiments [5, 6] have focused on only a single, modest speed of running (3.0 m/s); however, the effects of running speed on lateral balance are not known. We quantified how changes in running speed up to maximum sprint speed affect both ML foot placement and ML foot placement variability in non-amputee sprinters and sprinters with transtibial amputations. Since we were curious to understand how speed might affect one’s ability to properly modulate ML foot placement from step-to-step, we present and discuss this data in the text. However, our hypothesis concerns only ML foot placement variability—our indicator of lateral balance. We interpret our results with respect to a hypothesized relation between ML foot placement variability and lateral balance. The rationale for our hypothesis was that in order to attain faster running speeds, an individual must elicit briefer ground contact times and more rapid leg swings [1, 3, 12]. Because there is less time to adjust ML foot placement from step-to-step, it is likely that maintaining lateral balance becomes more challenging. We hypothesized that ML foot placement variability would increase across running speed up to maximum sprint speed in sprinters with and without transtibial amputations. We further hypothesized that ML foot placement variability would be symmetrical between the right and left legs of non-amputee sprinters but asymmetrically greater for the affected leg compared to the unaffected leg of sprinters with a unilateral transtibial amputation.

## Materials and Methods

### Subjects

Twelve non-amputee recreational sprinters and seven elite Paralympic sprinters with transtibial amputations (6 unilateral and 1 bilateral) volunteered to participate in this study. We selected non-amputee recreational sprinters who had similar maximal speeds to the Paralympic sprinters. The study was reviewed and approved by the Intermountain Healthcare Urban Central Region Institutional Review Board before the study began. Prior to experimental data collection, each subject read and signed the study’s written informed consent document. All data were collected at the Biomechanics Laboratory of the Orthopedic Specialty Hospital (Murray, Utah).

### Anthropometric Measurements

We measured height and body mass. The body mass of subjects with transtibial amputations included their running-specific prosthesis and socket mass (see S1 Table, which details the anthropometric and biomechanical characteristics for each sprinter that participated in this study).

### Experimental Protocol

**Figure 1:**
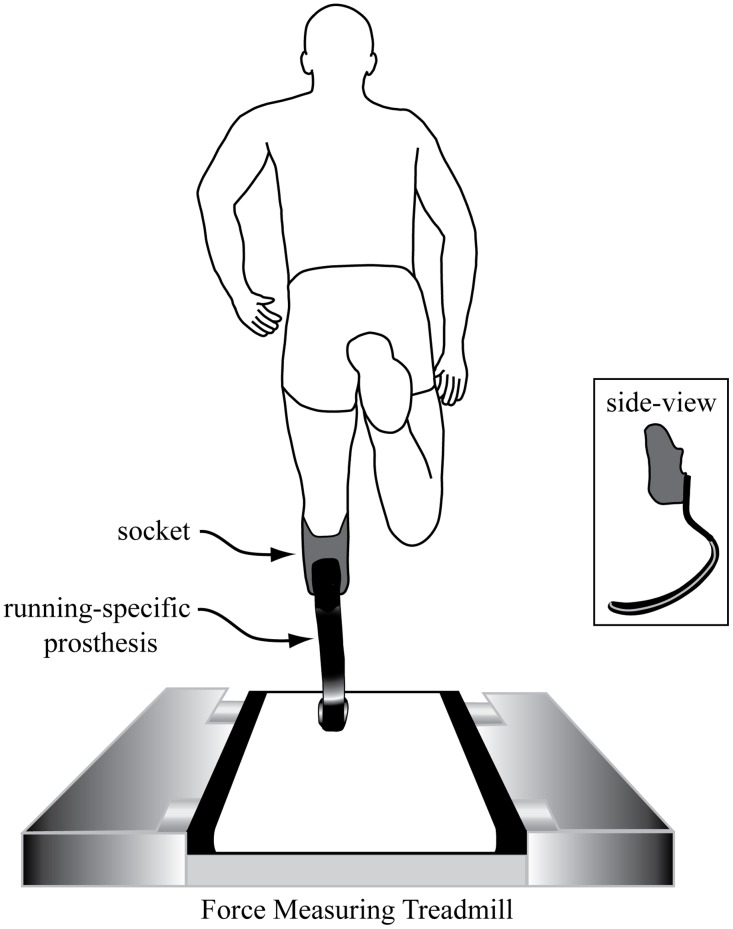
(a) Rear view of a sprinter with a left-side transtibial amputation running on a force-measuring treadmill. A side-view illustration of the running-specific prosthesis is provided as an inset. For measurements of ML foot placement relative to the body’s midline, we measured the position of the center of pressure at peak vertical ground reaction force for both biological legs and the legs using the running-specific prosthesis.

After emulating their pre-race warm-up (i.e. jogging, stretching, and short sprints), each subject performed brief running trials on a high-speed 3D force-sensing motorized treadmill (Fig. 1; Treadmetrix, Park City, UT). The treadmill is a custom designed lightweight treadmill with 3D force transducers (MC3A AMTI, Watertown, MA) at each corner that interface with amplifiers (MSA6 AMTI, Watertown, MA). During each trial, we simultaneously collected 3D ground reaction forces and moments (2400 Hz) and whole body kinematics from the 3D positions of reflective markers placed on the body (200 Hz; Motion Analysis Corporation, Santa Rosa, CA). We used a full-body custom marker set to define the position of the subject’s head, trunk, arms, legs, and running-specific prosthesis. Each subject began the series of trials at 3 m/s and we incremented the speed by 1 m/s until subjects approached their maximum speed. We then used smaller speed increments until the subject reached maximum speed, defined as the fastest speed at which the subject could maintain the same position on the treadmill for at least 20 consecutive steps. All subjects had experience with treadmill running and sprinting and were familiar with this task. During each trial, subjects lowered themselves from handrails onto the treadmill belt, which was moving at the testing speed. Handrails were placed along the front and sides of the treadmill and each subject had practice holding and then releasing the handrails when achieving maximum treadmill sprinting speeds (for clarity, handrails are not shown in Fig. 1).

### Data Analysis

**Figure 2:**
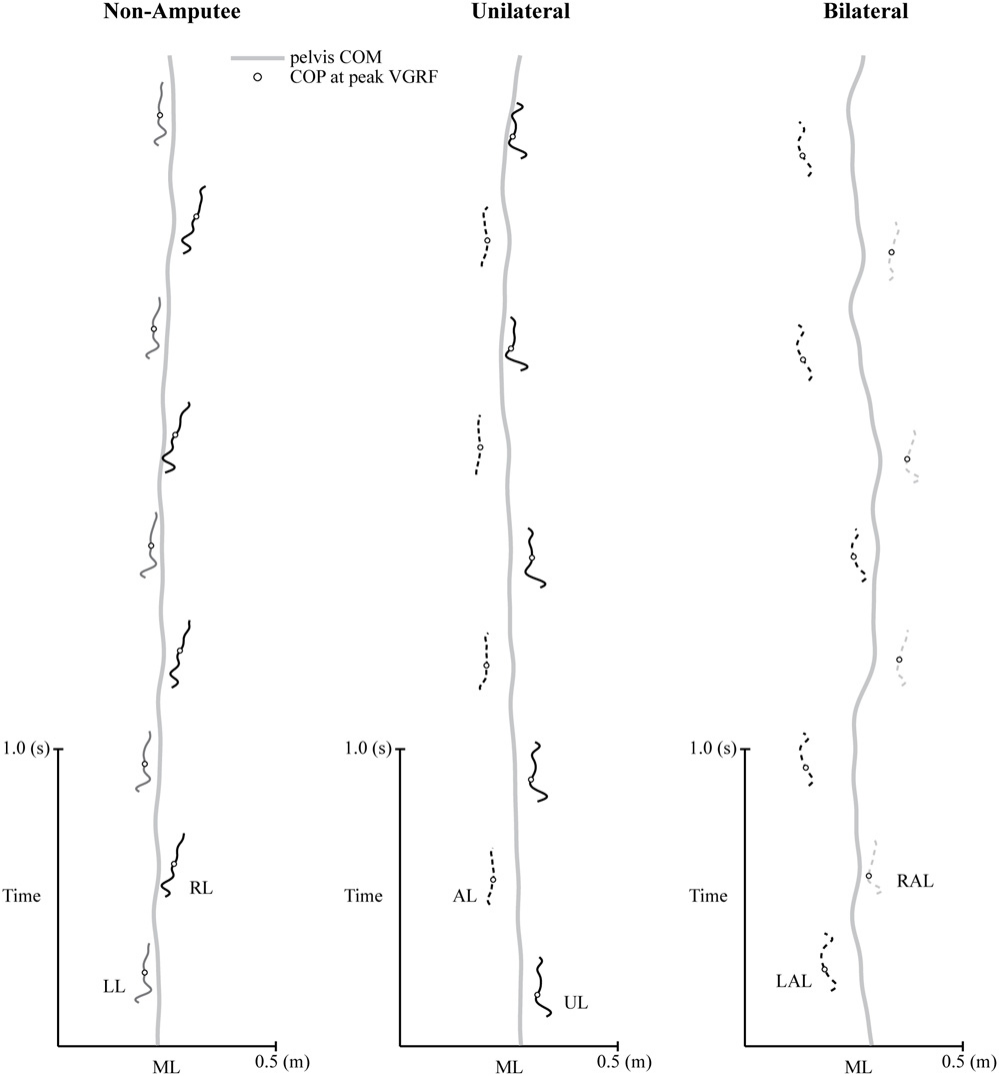
Representative time-series trajectories for the pelvis center of mass (COM) and center of pressure (COP) trajectories while running at 3 m/s for a non-amputee sprinter (Non-Amputee), a sprinter with a unilateral transtibial amputation (Unilateral), and a sprinter with bilateral transtibial amputations (Bilateral). For clarity, we use acronyms to denote the right leg (RL), left leg (LL), affected leg (AL), unaffected leg (UL), left affected leg (LAL), and right affected leg (RAL) for each respective sprinter. The ML position of the COP (open circles) indicates the instance of peak vertical ground reaction force (VGRF) during mid support for each step while running. Note that the Bilateral sprinter exhibits greater variation in ML foot placement from step-to-step when compared to the Non-Amputee and Unilateral sprinter.

We measured ML foot placement relative to the midline of the body and its variability from step-to-step using center of pressure (COP) data calculated from the force measuring treadmill (Fig. 2). Net ground reaction forces and COP were calculated using a calibration matrix and treadmill dimensions provided from the manufacturer. Force and COP signals were low pass filtered (4^th^ order, recursive Butterworth filter) at 20 Hz, 20 Hz, and 25 Hz for the AP, ML, and Vertical direction, respectively. The COP obtained from the treadmill was validated during a separate procedure by comparing the value to a known point of force application during running. This was accomplished by running with a rigid-sole shoe with a small bolt screwed to the bottom of the sole resulting in a contact point that was 1 cm in diameter. Markers were placed on the top of the shoe and on the contact point of the bolt. Once the location of the point was determined relative to the shoe markers, the marker on the contact point was removed. Our results show that while running at 3.1 m/s, the COP at peak vertical ground reaction force (~1600 N) differed from the contact point by 0.6 ± 1.5 mm in the ML direction.

Following a similar convention as McClay and Cavanagh [13], we defined the midline of the body as the ML position of the pelvis center of mass (COM*p*). The COM*p* was modeled as an elliptical cylinder [14] with dimensions defined by markers placed bilaterally on the iliac crests, anterior-superior iliac spines, greater trochanters, posterior-superior iliac spines, and sacrum. The location of the COM*p* was defined as the geometric center of the cylinder (Visual 3D, C-Motion Inc., Germantown MD.). We defined ML foot placement relative to the midline of the body as the distance between the COP and the COM*p* at the instance of the peak vertical ground reaction force that occurs during mid support for each step while running [15]. Our sign convention is that placing the left foot to the left of the midline is considered a positive foot placement, to the right of the midline a negative foot placement, placement on the line as zero, and vice versa.

Each subject performed each trial once. To control for the number of steps and to avoid bias in our variability measurements, we calculated the average ML foot placement during 20 consecutive steps that occurred during the middle of each trial. ML foot placement variability was defined as the standard deviation. From a total of 153 trials, we discarded 19 trials because they consisted of fewer than 20 consecutive steps or there was a large amount of marker drop out that could not be sufficiently corrected with gap filling and/or filtering techniques. Some of the discarded trials included the maximum speed for 5 non-amputee sprinters and 2 sprinters with a unilateral amputation. While a greater number of steps would be ideal [5], athletes can only sprint at their maximum speed for a short duration. Thus, we were inherently limited by the number of steps that we could incorporate into our variability analysis.

### Statistical Analysis

We normalized speed to each subject’s maximum speed prior to making comparisons between the legs of the same individual. We chose to normalize speed in this manner so that we could evaluate the change in ML foot placement and its variability across individuals with different absolute sprinting capabilities. Therefore, using an individual’s relative speed allows one to compare between sprinters using a standard scale that ranges from 0.3 to 1.0, where 1.0 is maximal speed. In contrast, we chose not to normalize M-L foot placement and its variability for two reasons. First, we were interested in comparing our data in absolute units, making it convenient for the reader to interpret by using physical measurements that are easily understandable. Second, normalizing ML foot placement and its variability to a maximum value of 1.0 would not allow us to illustrate differences between individual sprinters. In this normalized, hypothetical scenario, the maximum normalized value for both sprinters would be equal to 1.0 and one might interpret this to mean that both sprinters exhibited similar foot placement variability values. The absolute values; however, might be drastically different. For example, the maximum variability in sprinter #1 and #2 might be 1.0 cm and 2.0 cm, respectively. Therefore, expressing ML foot placement and its variability in absolute units makes it clear that the variability was greater in sprinter #2.

With respect to ML foot placement and its variability, we performed separate repeated measures MANOVAs to compare between the right and left leg of the non-amputee group and the unaffected and affected leg of the group with a unilateral amputation. We defined the “leg” as the within subjects fixed factor and “normalized speed” as a covariate. We avoided performing a between subjects comparison for two reasons: 1. the sample sizes between groups were unequal (non-amputee: *n* = 12 vs. unilateral: *n* = 6) and 2. our analysis was focused on comparing two dependent regression lines [16]. For example, we were interested in testing whether the regression line for ML foot placement variability of the right leg vs. normalized speed was significantly different from the regression line for ML foot placement variability of the left leg vs. normalized speed in non-amputee sprinters. We followed the same approach for the sprinters with a unilateral amputation.

**Figure 3:**
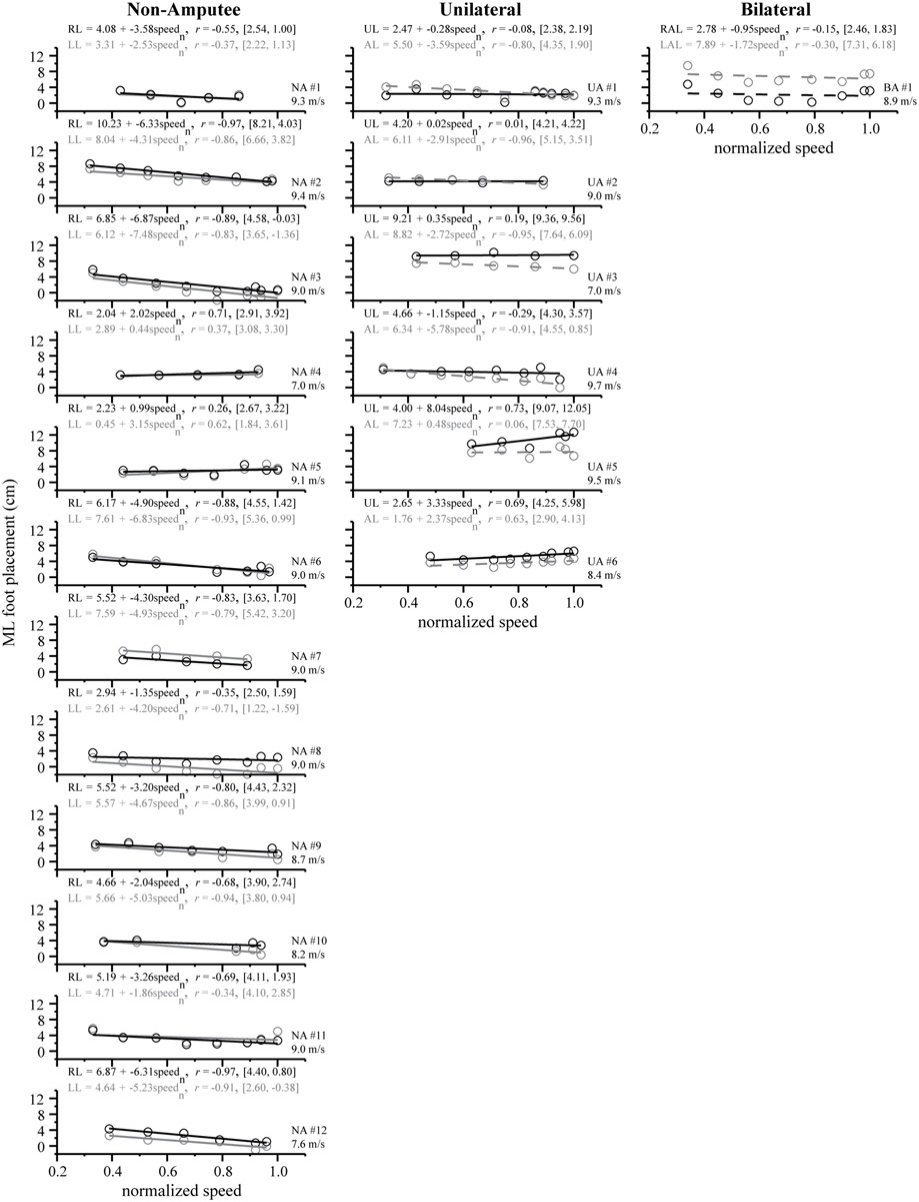
Individual linear regression lines for ML foot placement across normalized speed for non-amputee sprinters (Non-Amputee), sprinters with a unilateral transtibial amputation (Unilateral), and a sprinter with bilateral transtibial amputations (Bilateral). For clarity, we label each subplot with a “#” in the bottom, right corner that corresponds to each sprinter. Just below, we also include the absolute maximum speed (m/s) achieved by each sprinter. In general, non-amputee sprinters exhibited symmetrical changes in ML foot placement between the right leg (RL, black line) and left leg (LL, gray line) across normalized speed. In contrast to non-amputee sprinters, sprinters with a transtibial amputation exhibited varying degrees of asymmetry, as noted by differences in ML foot placement between the unaffected leg (UL, black line) and affected leg (AL, dashed gray line) and between the right affected leg (RAL, dashed black line) and left affected leg (LAL, dashed gray line). Note that for each sprinter’s leg, we present the regression equation, correlation coefficient, and range denoting the ML foot placement value achieved at minimum and maximum speeds.

**Figure 4:**
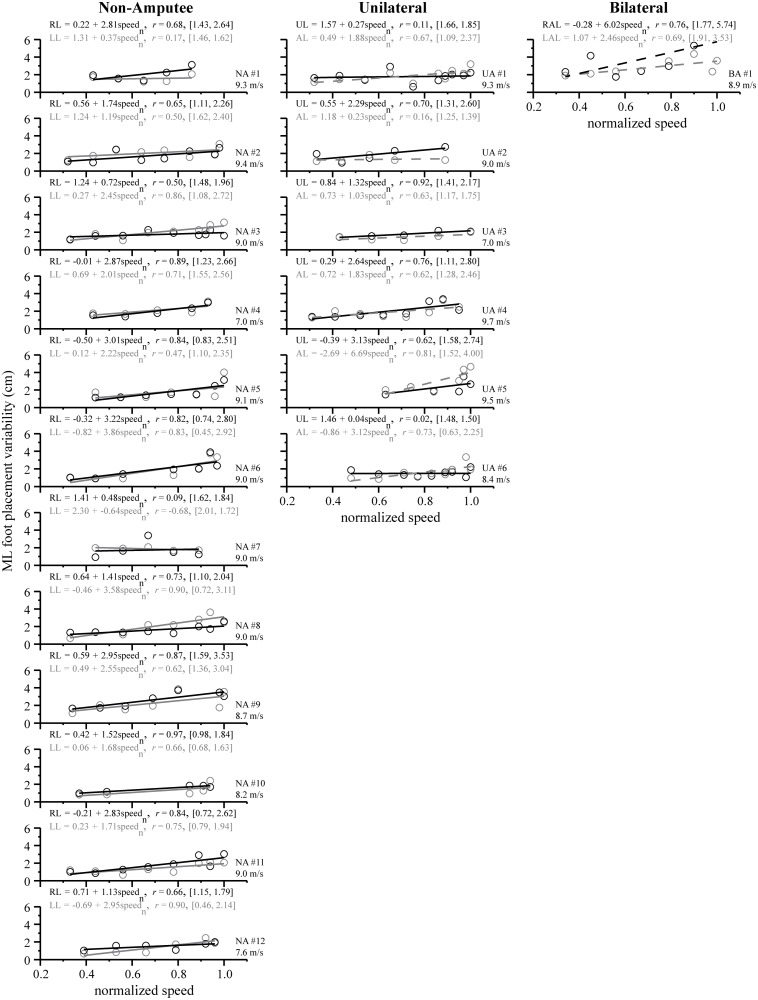
Individual linear regression lines for ML foot placement variability across normalized speed (graphical layout same as Fig. 3). In general, ML foot placement variability tended to increase across normalized speed, with the exception of the right leg (RL) and left leg (LL) of one non-amputee sprinter (#7) and the unaffected leg (UL) of one sprinter with a unilateral amputation (#6). Non-amputee sprinters also exhibited symmetrical changes in ML foot placement variability between the RL and LL across normalized speed. Similar to the ML foot placement trends (Fig. 3), sprinters with a transtibial amputation also exhibited varying degrees of asymmetry in ML foot placement variability, as illustrated by differences between the unaffected leg (UL) and affected leg (AL) and between the right affected leg (RAL) and left affected leg (LAL). Note that for each sprinter’s leg, we present the regression equation, correlation coefficient, and range denoting the ML foot placement variability value achieved at minimum and maximum speeds.

**Figure 5:**
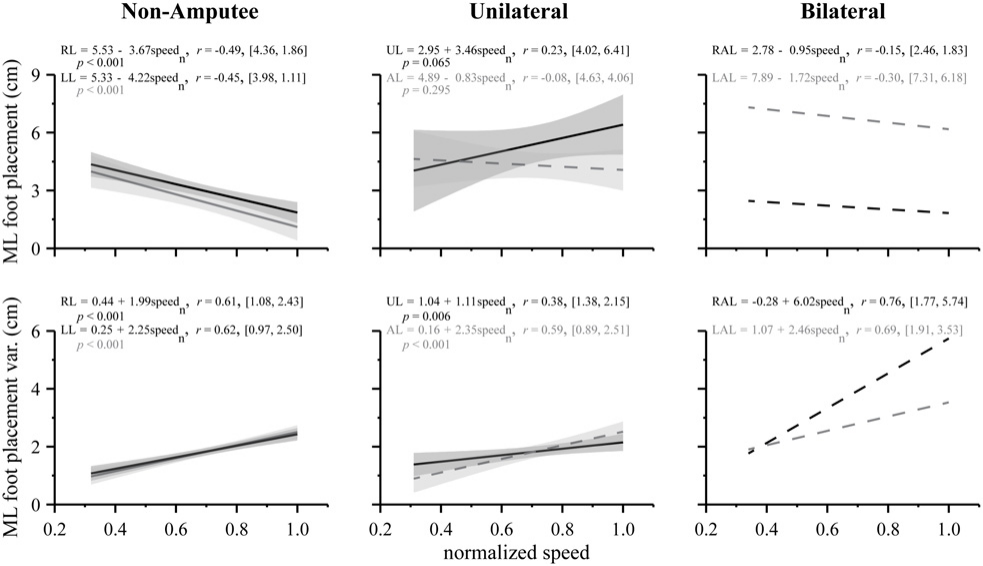
Average linear regression lines for ML foot placement (top) and ML foot placement variability (bottom) across normalized speed. Across speed, non-amputees exhibited symmetrical foot placement patterns between their right leg (RL) and left leg (LL). As non-amputee sprinters approached maximum speed, they placed their feet closer to the midline of the body; however, each leg exhibited greater ML foot placement variability (left column). In contrast, sprinters with unilateral transtibial amputations exhibited asymmetrical foot placement patterns between the affected leg (AL) and unaffected leg (UL). They tended to place the AL closer to the midline as compared to the UL, but the AL exhibited greater ML foot placement variability (middle column). The sprinter with bilateral transtibial amputations also exhibited asymmetrical foot placement patterns. Although he tended to place the right affected leg (RAL) closer to the midline of the body as compared to the left affected leg (LAL), ML foot placement was similar across speed. Both the LAL and RAL exhibited dramatic increases in ML foot placement variability at faster running speeds up to maximum sprint speed (right column). For each variable, we present the equation and *r* values from a least-squares linear regression analysis that included the individual data points across normalized speed for each sprinter. For the Non-Amputee and Unilateral group, the shaded regions represent the 95% lower and upper confidence bands. Since *n* = 1, we do not include the confidence bands for the Bilateral case. For clarity, individual data points are not shown.

To determine the direction and strength of the relation between variables, we followed the repeated measures MANOVAs with separate linear regression and correlation analyses for each dependent variable (i.e. ML foot placement and ML foot placement variability) with normalized speed as the independent variable. Linear regression and correlation (Pearson’s *r*) analyses were performed separately for non-amputee sprinters (*n* = 12) and sprinters with a unilateral amputation (*n* = 6). To elucidate the individual and group trends between ML foot placement, ML foot placement variability, and normalized speed, we present the linear regression equations denoting the slope, intercept, Pearson’s *r* values, and range (Figs. 3, 4, and 5). The range is defined in brackets, expressed in units of centimeters, and denotes the ML foot placement (or its variability) value at the individual’s minimum and maximum normalized speed. Statistical significance for all analyses was set at an *α* level = 0.05 (SPSS Inc., Chicago, IL).

It is important to note that the manner in which we incrementally increased running speed as we approached a sprinter’s maximum speed resulted in more data points closer to a normalized speed of 1.0 (see Figs. 3 and 4). Thus, the data are not evenly spread across normalized speed, which means that the overall magnitude and direction of the correlation coefficient may be heavily biased by the data points closer to 1.0. To be cautious that the strength of the correlation coefficients was not dramatically influenced by this phenomenon, we systematically compared the magnitude of the correlation coefficient with and without the cluster of data points. Although this resulted in more evenly spread data points across normalized speed, we found that the magnitude and direction of the correlation coefficients were roughly the same, therefore, we decided to include all the data in our individual and average regression analyses. Furthermore, since maximum speed data for some individuals was not available, we note that our regression analyses should be interpreted with this limitation in mind.

For our single sprinter with bilateral transtibial amputations, we report the regression equation, correlation values, and range to demonstrate the relation between the dependent variables and normalized speed. Because *n* = 1, we did not attempt statistical inference.

Since we were interested in highlighting any notable asymmetries that exist between the individual legs, we analyze and present individual as well as group outcomes. All the data underlying the findings in this study are freely available in the S1 Appendix.

## Results

### Non-Amputee Sprinters (n = 12)

**Individual analyses.** As indicated by the negative magnitude of the Pearson’s *r* values (Fig. 3, left column), 10 of 12 non-amputee sprinters exhibited a 0.9–4.6 cm decrease in ML foot placement with a two-fold increase in normalized speed. Two non-amputee sprinters (#4 and #5) did not decrease ML foot placement with speed. With respect to ML foot placement variability, 6 of 12 non-amputee sprinters exhibited an increase of 0.2–1.7 cm across the two-fold increase in normalized speed. The other 6 non-amputee sprinters exhibited a 1.4–2.1 cm increase in ML foot placement variability. Both of these trends are indicated by the positive magnitude of the Pearson’s *r* values (Fig. 4, left column).

**Group analyses.** Our regression analyses revealed that as non-amputee sprinters ran faster, they placed each foot closer to the body’s midline (*r*_RL_ = -0.50, *p* < 0.001 and *r*_LL_ = -0.45, *p* < 0.001; Fig. 5). At the slowest speed, non-amputee sprinters placed their feet ~ 4.1 cm lateral to the midline, whereas at maximum speed, they placed their feet 1.5 cm lateral to the midline, a 65% reduction. In contrast, ML foot placement variability of the right leg and left leg increased by 152% at maximum speed, compared to the slowest speed (*r*_RL_ = 0.61, p < 0.001 and *r*_LL_ = 0.62, *p* < 0.001). The average regression lines quantifying the changes in ML foot placement and ML foot placement variability across the two-fold increase in normalized speed were not significantly different between the right and left leg (Table 1, *p* = 0.569). As illustrated in Fig. 5 (left column), the average regression lines for ML foot placement and its variability for the right and left leg are similar in slope and intercept.

**Table 1:** Mean, standard deviation, and linear regression results based on a repeated measures MANOVA^a^ comparing ML foot placement and ML foot placement variability across normalized speed for the Non-Amputee group (*n* = 12).

**Main Effect: *leg***							
		**RL**		**LL**			
		Mean	SD	Mean	SD	Univariate ANOVA	*p* value
ML foot placement, cm		2.9	1.6	2.4	2.1	–	–
ML foot placement variability, cm		1.8	0.7	1.8	0.8	–	–
Interaction Effect: leg*normalized speed							
		Slope	Intercept	Slope	Intercept	Univariate ANOVA	*p* value
ML foot placement, cm		-3.67	5.53	-4.22	5.33	–	–
ML foot placement variability, cm		1.99	0.44	2.25	0.25	–	–

^**a**^A one-way repeated measures MANOVA with normalized speed as a covariate revealed no significant main effect for “leg”, indicating that ML foot placement and ML foot placement variability between the RL and LL was not statistically different: *F*_2,77_ = 0.43, *p* = 0.650, Wilk’s λ = 0.99. In addition, our statistical analysis revealed no significant interaction effect between “leg” and “normalized speed”, indicating that the dependent regression lines quantifying the linear relation of ML foot placement or ML foot placement variability across normalized speed for the RL and LL were not statistically different: *F*_2,77_ = 0.57, *p* = 0.569, Wilk’s λ = 0.98. Since significance was not detected, we did not follow up with univariate ANOVAs.

### Sprinters with Unilateral Amputations (n = 6)

**Individual analyses.** We found that changes in ML foot placement across normalized speed were different between the unaffected and affected leg of sprinters with unilateral amputations (Fig. 3, middle column). Unilateral sprinters #1, 2, 3, and 4 exhibited a 1.6–3.7 cm decrease in ML foot placement of the affected leg (*r*_AL_ = -0.80 to -0.95) while maintaining the same position of the unaffected leg (*r*_UL_ = -0.01 to -0.29) across normalized speed. In contrast, unilateral sprinter #5 exhibited a 3.0 cm increase in ML foot placement of the unaffected leg (*r*_UL_ = 0.73) while maintaining the same position of the affected leg (*r*_AL_ = 0.06). Only one unilateral sprinter (#6) showed similar increases in ML foot placement for both their unaffected leg (1.7 cm) and affected leg (1.2 cm) across normalized speed, although the unaffected leg was consistently placed further away from the midline.

We also found that changes in ML foot placement variability across normalized speed were different between the unaffected and affected leg. In unilateral sprinters #1 and 6, the variability of the unaffected leg remained the same while the variability of the AL exhibited a 1.3 cm increase across normalized speed (Fig. 4, middle column). In unilateral sprinter #2, the variability of the unaffected leg exhibited a 1.3 cm increase while the variability of the affected leg remained the same across normalized speed. In unilateral sprinter #3, the variability of both the unaffected and affected leg exhibited a less than 1 cm increase across normalized speed. In unilateral sprinter #4, the unaffected and affected leg exhibited a 1.7 cm and 1.2 cm increase in variability across normalized speed, respectively. In unilateral sprinter #5, the variability of the unaffected leg exhibited a 1.2 cm increase while the variability of the affected leg exhibited a 2.5 cm increase across normalized speed. To emphasize these dramatic asymmetries a bit further, the Pearson’s *r* values for the affected leg in three unilateral sprinters (#1, 5, and 6) was notably higher than the unaffected leg, indicating that across normalized speed, the variability of the affected leg increased to a greater extent than the unaffected leg. In contrast, the Pearson’s *r* value for the unaffected leg in one unilateral sprinter (#2) was notably higher than the affected leg, indicating that across normalized speed, the variability of the unaffected leg increased to a greater extent than the affected leg. Unilateral sprinters #3 and #4 showed similar increases in the variability of the unaffected and affected legs across normalized speed. For unilateral sprinter #3, however, the variability of the unaffected leg was consistently higher than the AL.

**Group analyses.** In contrast to non-amputees, there was no significant relation for ML foot placement of the unaffected or affected leg across normalized speed (*r*_UL_ = 0.235, *p* = 0.065 and *r*_AL_ = -0.085, *p* = 0.295; Fig. 5, middle column), although the regression lines were significantly different between the unaffected and affected leg (*p* < 0.001). ML foot placement variability of the unaffected and affected leg increased across normalized speed; however, the regression lines were statistically different in slope and intercept (Table 2, *p* = 0.020). As indicated by the slopes, a unit increase in normalized speed coincides with an ~ 1 cm and 2 cm increase in ML foot placement variability of the unaffected and affected leg, respectively. Most notably, the unaffected and affected leg exhibited a 57% and 190% increase in ML foot placement variability, respectively (*r*_UL_ = 0.377, *p* = 0.006 and *r*_AL_ = 0.586, *p* < 0.001).

**Table 2:** Mean, standard deviation, and linear regression results based on a repeated measures MANOVA^b^ comparing ML foot placement and ML foot placement variability across normalized speed for the Unilateral group (*n* = 6).

**Main Effect: *leg***							
		**AL**		**UL**			
		Mean	SD	Mean	SD	Univariate ANOVA	*p* value
ML foot placement, cm		4.3	2.1	5.5	3.2	*F*_1,41_ = 6.77	0.013
ML foot placement variability, cm		1.9	0.9	1.8	0.6	*F*_1,41_ = 5.02	0.030
Interaction Effect: leg*normalized speed							
		Slope	Intercept	Slope	Intercept	Univariate ANOVA	*p* value
ML foot placement, cm		-0.83	4.89	3.46	2.95	*F*_1,41_ = 19.07	< 0.001
ML foot placement variability, cm		2.35	0.16	1.11	1.04	*F*_1,41_ = 5.83	0.020

^**b**^A one-way repeated measures MANOVA with normalized speed as a covariate revealed a significant main effect for “leg”, indicating that ML foot placement and ML foot placement variability between the AL and UL were statistically different: *F*_2,40_ = 5.10, *p* = 0.011, Wilk’s λ = 0.80. In addition, our statistical analysis revealed a significant interaction effect between “leg” and “normalized speed”, indicating that the dependent regression lines quantifying the linear relation of ML foot placement or ML foot placement variability across normalized speed for the AL and UL were statistically different: *F*_2,40_ = 10.99, *p* < 0.001, Wilk’s λ = 0.65 Since significance was detected, we followed up with univariate ANOVAs.

### Sprinter with Bilateral Amputations (n = 1)

Similar to sprinters with a unilateral amputation, we found that our regression analyses did not reveal strong trends between ML foot placement of the right or left affected leg and normalized speed in the sprinter with bilateral amputations (*r*_RAL_ = -0.15 and *r*_LAL_ = -0.30; Fig. 5, right column). However, ML foot placement variability of the right affected leg exhibited a 4.0 cm increase while the left affected leg exhibited a 1.6 cm increase across normalized speed. When comparing his maximum speed to his slowest speed of 3 m/s, the bilateral sprinter exhibited a 276% and 95% increase in ML foot placement variability for the right and left affected leg, respectively (*r*_RAL_ = 0.76 and *r*_LAL_ = 0.69). Across normalized speed, ML foot placement of the left affected leg (6.7 ± 1.4 cm) was substantially greater than the right affected leg (2.1 ± 1.6 cm) but both ML foot placements were within the range observed in sprinters with a unilateral transtibial amputation. ML foot placement variability of the right affected leg (4.0 ± 2.0 cm) was slightly greater than the left affected leg (2.8 ± 0.9 cm) but was on average 89% greater than the affected leg of sprinters with a unilateral amputation.

## Discussion

Our data support our 1^st^ hypothesis that ML foot placement variability in sprinters with and without transtibial amputations generally increases with running speed up to maximum sprint speed. Our data also support our 2^nd^ hypothesis that ML foot placement variability is symmetrical between the right and left legs of non-amputee sprinters and asymmetrically greater for the affected leg (with a running specific prosthesis) compared to the unaffected leg of sprinters with a unilateral transtibial amputation. Upon close inspection, we also found that increases in ML foot placement variability across speed differed between the affected and unaffected leg, highlighting the fact that the legs of each sprinter with a unilateral amputation showed a modest, yet asymmetrical response to increases in running speed up to maximum speed.

### Modulation of ML foot placement across speed

Although we did not attempt to statistically compare between groups, we would be remiss, given the unique nature of our data set, if we did not make some general observations. Overall, all sprinters adopted a positive foot placement while running and sprinting, i.e. placing the foot lateral to the body’s midline. As they approached maximum speed, non-amputee sprinters tended to place their feet closer to the body’s midline. On the contrary, sprinters with unilateral or bilateral transtibial amputations did not show a systematic tendency to place their feet closer to the midline. One explanation for this could be that modulating foot placement across speed might be difficult for sprinters with an amputation because they lack tactile exteroreception and proprioception in their affected leg. Indeed, a combination of afferent input arising from the leg and foot plays an important role in proper foot placement during running, which is required for controlling the body’s center of mass trajectory [17]. Another possibility is that sprinters with a unilateral amputation place each foot slightly away from the midline to compensate for inertial and ground force asymmetries that exist between the affected and unaffected legs [1]. For the sprinter with bilateral transtibial amputations, ML foot placement between the right and left affected leg exhibited the highest asymmetry across speed. For the same reasons mentioned above, it seems reasonable to postulate that the loss of proprioception and fine muscular control in both legs would make it more difficult to modulate foot placement from step-to-step.

### Increase in ML foot placement variability across speed

In general, a steady increase in speed coincided with steady increases in ML foot placement variability. This modest, but steady increase in ML foot placement variability suggests that maintaining lateral balance becomes more challenging at faster running speeds up to a maximum normalized sprint speed of 1.0 [5, 6]. While the increase in ML foot placement variability between the right and left legs was similar for non-amputee sprinters, the variability of the affected leg in sprinters with a unilateral amputation generally increased to a greater extent than the unaffected leg across the two-fold increase in normalized speed (Fig. 5). However, one must be cautious in generalizing these average regression trends because the changes in our foot placement data were highly variable between sprinters with a unilateral transtibial amputation. For example, ML foot placement variability of the affected leg increased more dramatically than the unaffected leg across normalized speed for 3 out of the 6 sprinters with a unilateral amputation. The major finding to take away from our average regression analysis is that increases in ML foot placement variability across speed were asymmetric between the affected and unaffected leg.

### Symmetry vs. Asymmetry

As expected, we found similar changes in ML foot placement and its variability between the right and left legs across speed, reflecting a high degree of symmetry between the biological legs of non-amputee sprinters. Statistically speaking, the average correlation coefficient expressing the strength of the linear trend between ML foot placement variability and normalized speed (*r* = 0.61) falls within a moderate to large magnitude [18], suggesting a moderate to substantial effect of increased speed on increased ML foot placement variability. In contrast, the large differences in the values of the correlation coefficients (*r*_UL_ vs. *r*_AL_) demonstrate that changes in ML foot placement and its variability across speed were asymmetric between the affected and unaffected leg of sprinters with a unilateral transtibial amputation. Previous studies of individuals with a transtibial amputation have identified important asymmetries in sagittal plane kinematics and kinetics while running and sprinting [1, 2, 7, 8, 10, 11]. Here, we demonstrate that sprinters with a unilateral transtibial amputation also exhibit biomechanical asymmetries in the frontal plane. In general, however, the average increase in ML foot placement variability across speed in sprinters with a unilateral amputation fell within the range observed in non-amputee sprinters. Our data suggest that when running at faster speeds up to maximum sprint speed, sprinters with a unilateral transtibial amputation found maintaining lateral balance just as challenging as non-amputee sprinters.

Perhaps our most intriguing data are from the sprinter with bilateral transtibial amputations, who exhibited the greatest increases in ML foot placement variability with speed. Our supplementary videos provide an immediate and intuitive appreciation of our empirical data (see S1–S12 Videos, which demonstrates a non-amputee sprinter, a sprinter with a unilateral transtibial amputation, and a sprinter with bilateral transtibial amputations running at their slowest speed and sprinting at their maximum speed). Video representations of the running and sprinting kinematics of these sprinters in the sagittal plane (side-view) appear very similar. However, it is only when one examines the running and sprinting performance from the frontal plane (rear-view) that obvious differences visually emerge in ML foot placement from step-to-step. This affect can be appreciated when comparing the sprinter with bilateral transtibial amputations to a non-amputee sprinter and a sprinter with a unilateral transtibial amputation. When compared to all other sprinters in this study, our data suggest that the sprinter with bilateral transtibial amputations experienced the greatest challenge with maintaining lateral balance, especially at his maximum sprint speed. However, we interpret these findings with caution because we only collected data on one elite sprinter with bilateral transtibial amputations who at the time had only two years of experience using running-specific prostheses. In our future work, we hope to investigate ML foot placement and its variability in additional sprinters with bilateral transtibial amputations and to determine if more experience with the use of running-specific prostheses improves the ability to modulate foot placement from step-to-step.

Although we use measurements of ML foot placement variability as an indicator of lateral balance, some may consider our simple approach to be a limitation. We recognize that foot placement variability measures alone do not quantify dynamic stability nor do they provide rigorous insights into the control of balance from a first-principles approach. We emphasize here that we did not attempt to quantify dynamic stability or undertake a first-principles approach. Although it was beyond the scope of our present analyses, we have recently pursued nonlinear measures of dynamic stability that have been previously used to study human running [19]. In brief, we quantified the maximal Lyapunov exponent (LyE) from the leg joint dynamics of sprinters without and with a unilateral transtibial amputation [20]. We found that the maximal LyE increased as running speed increased, indicating that sprinters were less stable as running speed increased. Furthermore, while dynamic stability was similar between the biological legs of non-amputee sprinters, we found that the dynamics of the affected leg were less stable than the unaffected leg in sprinters with a unilateral transtibial amputation, revealing notable asymmetries. Taken together, our simple foot placement variability measures reveal similar trends as the relatively complex nonlinear measures of dynamic stability.

Other potential limitations of our study include our relatively small sample size, using the COM*p* as a proxy for the body’s midline, the use of a treadmill, and differences in maximum speed performances between subjects. Because we chose to focus on elite Paralympic sprinters, our sample size of sprinters with transtibial amputations was small, however, we believe that our unique population allowed us to understand how the use of running-specific prosthesis affects ML foot placement and its variability. We used the COM*p* to define the body’s midline, but ideally would have measured ML foot placement relative to the whole body COM (COM*wb*). We did not attempt to calculate COM*wb* because of potential inaccuracies in modeling the running-specific prostheses used by the sprinters with transtibial amputations. We found that the COM*p* is a good reference for the body’s midline, and moreover, using the COM*p* as the body’s midline kept the point of reference the same for all groups. While there may be differences between the two (COM*p* vs. COM*wb*), they are likely small and consistent in the ML direction, thereby having little influence on our results. All subjects had experience with treadmill running and sprinting prior to participating in the experiment, but differences in ML foot placement and its variability may exist between treadmill and over-ground running and sprinting. In addition, as opposed to using a counterbalanced or random design, it is possible that incrementally increasing treadmill speed up to each sprinters maximum speed might have biased our results. To minimize these potential effects, we allowed subjects a full recovery *ad libitum* following each bout of running or sprinting. Finally, the training and best performances of these athletes may be relevant to one’s ability to properly modulate ML foot placement from step-to-step and as an important consequence, lateral balance. As noted in Figs. 3 and 4, the maximum speed achieved by each sprinter ranged from 7.0–9.7 m/s. It would be worthwhile in the future to compare athletes based on different levels of sprinting experience and training background. Nonetheless, we feel that the maximum sprint speed performances of all athletes in this experiment gave an accurate description of their sprinting ability.

## Conclusions

Because the use of running-specific leg prostheses are becoming more common, measures of ML foot placement and its variability may be useful for those seeking simple metrics to assess potential balance problems in runners and sprinters. Overall, our study highlights several important findings about sprinters with and without transtibial amputations. We infer from our results that 1) when compared to slow speeds, maintaining lateral balance is more challenging at faster running speeds up to maximum sprint speed and 2) sprinters with a unilateral transtibial amputation found maintaining lateral balance just as challenging as non-amputee sprinters. Furthermore, the apparent asymmetries in ML foot placement and its variability suggest that the use of running-specific prostheses results in a compensatory foot placement strategy for maintaining lateral balance in sprinters with a unilateral transtibial amputation. Finally, when compared to all other sprinters in our subject pool, the sprinter with bilateral transtibial amputations exhibited the greatest increases in ML foot placement variability across normalized speed, indicating that maintaining lateral balance was the most challenging for this Paralympic sprinter.

## Supporting Information

S1 Table Table that details the anthropometric and biomechanical characteristics for each sprinter that participated in this study. (DOCX)

S1 Appendix The data underlying the findings in this study. (XLS)

S1 Video Video from the side-view showing a non-amputee sprinter at his slowest speed of 3.0 m/s. (MOV)

S2 Video Video from the rear-view showing a non-amputee sprinter at his slowest speed of 3.0 m/s. (MOV)

S3 Video Video from the side-view showing a non-amputee sprinter at his maximum speed of 9.0 m/s. (MOV)

S4 Video Video from the rear-view showing a non-amputee sprinter at his maximum speed of 9.0 m/s. (MOV)

S5 Video Video from the side-view showing a sprinter with a unilateral transtibial amputation at her slowest speed of 3.0 m/s. (MOV)

S6 Video Video from the rear-view showing a sprinter with a unilateral transtibial amputation at her slowest speed of 3.0 m/s. (MOV)

S7 Video Video from the side-view showing a sprinter with a unilateral transtibial amputation at her maximum speed of 9.0 m/s. (MOV)

S8 Video Video from the rear-view showing a sprinter with a unilateral transtibial amputation at her maximum speed of 9.0 m/s. (MOV)

S9 Video Video from the side-view showing a sprinter with bilateral transtibial amputations at his slowest speed of 3.0 m/s. (MOV)

S10 Video Video from the rear-view showing a sprinter with bilateral transtibial amputations at his slowest speed of 3.0 m/s. (MOV)

S11 Video Video from the side-view showing a sprinter with bilateral transtibial amputations at his maximum speed of 8.9 m/s. (MOV)

S12 Video Video from the rear-view showing a sprinter with bilateral transtibial amputations at his maximum speed of 8.9 m/s. (MOV)
