# Supplementary material for: Effect of Running Speed and Leg Prostheses on Mediolateral Foot Placement and Its Variability
Source: PLoS One. 2015 Jan 15;10(1):e0115637. doi: 10.1371/journal.pone.0115637 (PMC4295868; doi:10.1371/journal.pone.0115637)
Supplement: S1 Table — (DOCX) [file pone.0115637.s001.docx]

Table S1. Anthropometric and biomechanical characteristics for each unilateral (UL), bilateral (BL), and non-amputee (NA) sprinter. For sprinters with unilateral and bilateral transtibial amputations, total mass includes prosthesis mass.

|  | sex  (M/F) | age (years) | height  (m) | total mass (kg) | UL  (or RL) length (m) | AL  (or LL) length (m) | RSP (model) | RSP mass (kg) |
| --- | --- | --- | --- | --- | --- | --- | --- | --- |
| 1UL | F | 35 | 1.68 | 66.1 | 0.955 | 0.985 | [Össur](http://www.ossur.com/?select-default-destination=1) Cheetah | 1.7 |
| 2UL | F | 23 | 1.69 | 62.9 | 0.895 | 0.950 | Ottobock Sprinter | 1.2 |
| 3UL | M | 36 | 1.84 | 79.9 | 0.985 | 1.030 | [Össur](http://www.ossur.com/?select-default-destination=1) Cheetah | 1.6 |
| 4UL | M | 27 | 1.75 | 69.3 | 0.930 | 0.970 | [Össur](http://www.ossur.com/?select-default-destination=1) Cheetah | 1.5 |
| 5UL | M | 29 | 1.87 | 109.1 | 0.985 | 1.065 | Ottobock Sprint-Run | 1.4 |
| 6UL | M | 25 | 1.85 | 71.0 | 1.005 | 1.025 | [Össur](http://www.ossur.com/?select-default-destination=1) Cheetah | 1.2 |
|  |  |  |  |  |  |  |  |  |
| 1BL | M | 20 | 1.75 | 67.7 | 0.995 | 1.005 | [Össur](http://www.ossur.com/?select-default-destination=1) Cheetah | 1.7 |
|  |  |  |  |  |  |  |  |  |
| 1NA | F | 16 | 1.70 | 59.5 | 0.900 | 0.900 | - | - |
| 2NA | F | 18 | 1.70 | 56.4 | 0.885 | 0.890 | - | - |
| 3NA | F | 28 | 1.74 | 60.9 | 0.952 | 0.945 | - | - |
| 4NA | M | 40 | 1.69 | 88.6 | 0.885 | 0.890 | - | - |
| 5NA | M | 16 | 1.75 | 69.1 | 0.920 | 0.935 | - | - |
| 6NA | M | 16 | 1.82 | 70.7 | 0.950 | 0.955 | - | - |
| 7NA | M | 19 | 1.88 | 89.1 | 0.990 | 1.000 | - | - |
| 8NA | M | 21 | 1.84 | 75.0 | 1.000 | 1.000 | - | - |
| 9NA | M | 18 | 1.79 | 76.8 | 0.910 | 0.915 | - | - |
| 10NA | M | 17 | 1.80 | 78.2 | 0.960 | 0.940 | - | - |
| 11NA | M | 18 | 1.80 | 73.2 | 0.905 | 0.915 | - | - |
| 12NA | M | 18 | 1.85 | 84.1 | 0.985 | 0.980 | - | - |
